# Unprecedented

**Published:** 1898-06

**Authors:** 


UNPRECEDENTED.—At the recent meeting of the Texas State Medical Association at Houston the "Red Back" was represented by a young lady solicitor, and the space in the vestibule of the hall occupied by JOURNAL and representative was that required for a small table. For that space the JOURNAL has received (and paid) a bill for $5, made out in the name of the Chairman of the Committee of Arrangements. This is an outrage. To what is the revenue expected or received from this source to be applied? To the expense of making arrangements, banquets, excursions, etc.? or to the expense of the Association? We want to know.

---

PETER PIPER PICKED a peck of pepper. His granny wanted it to put in her vegetable compound for the diarrhœa, or summer troubles to which her young 'uns were subject. That was in the long ago. Medicine in modern times is less disagreeable to take and more efficient. Better than Peter Piper's Pepper by far is *Peter's Peptic Essence Compound* (Arthur Peter & Co., Louisville). The time of year is here when old and young have intestinal troubles. P. P. E. Co. (Arthur Peter & Co.) contains the necessary digestive ferments; it acts in the stomach and in the small bowels, correcting the conditions that give rise to acid and watery actions and changes them to healthy movements. It enables a child to retain and assimilate food which usually runs through unchanged. Send for pamphlet. Mention this journal.

